# Tumour necrosis factor induces increased production of extracellular amyloid-β- and α-synuclein-containing aggregates by human Alzheimer’s disease neurons

**DOI:** 10.1093/braincomms/fcaa146

**Published:** 2020-09-15

**Authors:** Daniel R Whiten, Philip W Brownjohn, Steven Moore, Suman De, Alessio Strano, Yukun Zuo, Moritz Haneklaus, David Klenerman, Frederick J Livesey

**Affiliations:** 1 Department of Chemistry, University of Cambridge, Cambridge CB2 1EW, UK; 2 Zayed Centre for Research into Rare Disease in Children, UCL Great Ormond Street Institute of Child Health, London WC1N 1DZ, UK; 3 UK Dementia Research Institute at University of Cambridge, Cambridge CB2 0XY, UK

**Keywords:** inflammation, protein aggregation, tumour necrosis factor, induced pluripotent stem cells, presenilin-1

## Abstract

In addition to increased aberrant protein aggregation, inflammation has been proposed as a key element in the pathogenesis and progression of Alzheimer’s disease. How inflammation interacts with other disease pathways and how protein aggregation increases during disease are not clear. We used single-molecule imaging approaches and membrane permeabilization assays to determine the effect of chronic exposure to tumour necrosis factor, a master proinflammatory cytokine, on protein aggregation in human-induced pluripotent stem cell-derived neurons harbouring monogenic Alzheimer’s disease mutations. We report that exposure of Alzheimer’s disease neurons, but not control neurons, to tumour necrosis factor induces substantial production of extracellular protein aggregates. Aggregates from Alzheimer’s disease neurons are composed of amyloid-β and α-synuclein and induce significant permeabilization of lipid membranes in an assay of pathogenicity. These findings provide support for a causal relationship between two crucial processes in Alzheimer’s disease pathogenesis and suggest that targeting inflammation, particularly tumour necrosis factor, may have beneficial downstream effects on ameliorating aberrant protein aggregation and accumulation.

## Teaser

There is a complex interplay between neuroinflammation and protein aggregation in Alzheimer’s disease. We show that chronic exposure to the inflammatory cytokine tumour necrosis factor stimulates monogenic Alzheimer’s disease neurons to produce significantly more toxic protein aggregates, with no effect on healthy neurons, indicating a causal relationship between neuroinflammation and protein aggregation.

## Graphical Abstract

**Graphical Abstract:**
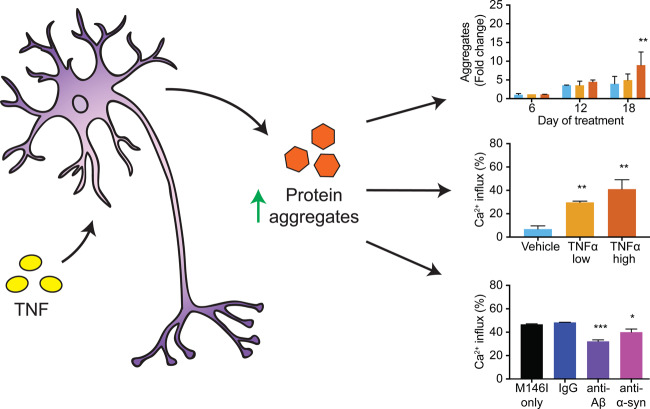

## Introduction

Accumulation of misfolded proteins produced by neurons, initially extracellular deposits of amyloid-β (Aβ), followed by intracellular aggregation of tau are thought to be primary initiating mechanisms in Alzheimer’s disease pathogenesis (Hardy and Higgins, 1992; Hardy and Selkoe, 2002). Downstream of this biochemical phase of Alzheimer’s disease are complex interactions between neurons and astrocytes, microglia and other cell types, resulting in chronic neuroinflammation, synaptic dysfunction and eventually widespread cell loss (De Strooper and Karran, 2016). While inflammation in this context has been thought of as a reactive and secondary process, clinical, genetic and experimental evidence now suggests a more central upstream role for immune system involvement in Alzheimer’s disease pathophysiology and progression [reviewed by Heneka *et al.* (2015)], although how this initiates is not clear.

Several lines of evidence support the hypothesis that inflammation, either from the disease itself, or derived from secondary insults, augments pathology and cognitive decline in Alzheimer’s disease. First, increased levels of central and systemic proinflammatory cytokines are observed in mild cognitive impairment and are positively correlated with progression into severe Alzheimer’s disease (Tarkowski *et al.*, 2003; Bermejo *et al.*, 2008). Second, central neuroinflammation as a result of traumatic brain injury speeds deposition of Alzheimer’s disease-associated proteins and cognitive impairment in animal models (Tran *et al.*, 2011; Tajiri *et al.*, 2013), while a putative epidemiological link has been established between traumatic brain injury and future risk for Alzheimer’s disease (Dams-O’Connor *et al.*, 2016). Finally, systemic inflammation is associated with higher levels of circulating proinflammatory cytokines and an increased rate of cognitive decline in Alzheimer’s disease patients (Dunn *et al.*, 2005; Holmes *et al.*, 2009) and accelerates primary neurodegenerative phenotypes, in particular protein aggregation and deposition, in animal models (Cunningham *et al.*, 2009; Kyrkanides *et al.*, 2011). The mechanisms underlying the relationship between a proinflammatory environment and primary disease phenotypes is yet to be fully elucidated, however.

We and others have reported that cortical excitatory neurons generated *in vitro* from induced pluripotent stem cells (iPSCs) derived from individuals carrying disease-causing mutations in amyloid precursor protein (*APP*) or presenilin 1 (*PSEN1*) display robust and reproducible alterations in extracellular Aβ species (Yagi *et al.*, 2011; Israel *et al.*, 2012; Moore *et al.*, 2015), increased tau levels and phosphorylation (Israel *et al.*, 2012; Moore *et al.*, 2015) and the production of synaptotoxic forms of Aβ and tau (Hu *et al*., 2018). Furthermore, neurons with mutations in *PSEN1* or *APP* have pronounced dysregulation of the endolysosome autophagy network (Hung and Livesey, 2018), with no evidence of intracellular protein aggregation. Modelling of these monogenic, early onset cases of Alzheimer’s disease in human cellular systems has the potential to inform our understanding of the more common yet complex late onset form of the disease, while allowing for reductionist approaches towards elucidating complex interactions between disease pathways in sensitized genetic backgrounds.

To investigate the relationship between inflammation and protein aggregation in Alzheimer’s disease, we exposed iPSC-derived Alzheimer’s disease neurons, which produce higher proportions of aggregation-prone Aβ peptides, to the proinflammatory cytokine tumour necrosis factor (TNF), a key mediator common to both central and systemic inflammatory pathways, and measured changes in the production and composition of extracellular aggregates of disease-associated proteins. Using ultrasensitive single-aggregate imaging techniques (Horrocks *et al.*, 2016; Whiten *et al.*, 2018b) and lipid permeabilization assays (Flagmeier *et al.*, 2017) capable of characterizing the low concentration of secreted aggregates, we show that long-term treatment with TNF substantially increases the production of biologically active Aβ and α-synuclein-containing aggregates specifically in compromised familial Alzheimer’s disease neurons, but not healthy neurons, suggestive of a role for inflammation in early Alzheimer’s disease pathology.

## Materials and methods

### Generation of human cortical neuron cultures

The iPSC lines used in this study were non-demented control and *PSEN1* Intron 4 and M146I, as previously reported (Moore *et al.*, 2015). Pluripotent cells were maintained on Geltrex in Essential 8 media (both Thermofisher). With minor modifications to account for feeder-free iPSC maintenance, directed differentiation to cerebral cortex was performed as previously described (Shi *et al.*, 2012). Briefly, confluent monolayers of iPSCs were induced to form neural progenitors over the course of 12 days by dual-SMAD inhibition. Cortical progenitors were enriched and then expanded between Days 12 and 35 post-neural induction before passaging onto Geltrex-coated plates at a density of 80 000 cells per cm^2^ for neuronal differentiation and maturation.

### TNF treatment

Treatments with recombinant human TNF at 0.1 and 10 ng/ml (Peprotech) or the vehicle control were started 60 days post-neural induction and continued for a duration of 18 days. During this period, a complete exchange of cell culture media was performed every 48 h. The secretomes of neuronal cultures were collected every 6 days, centrifuged at 800 RCF for 3 min to remove cellular debris and stored at −20°C until required for analysis. For washout experiments, cultures were switched from TNF treatment to the vehicle control after the collection of secretomes at Day 12.

### Multiplexed Aβ ELISA

Quantification of Aβ38, Aβ40 and Aβ42 was performed with the V-PLEX Aβ peptide Panel 1 kit (K15200E) and a Quickplex SQ120 instrument (MesoScale Discovery) using 25 µl of cell culture supernatant collected at Day 18 of TNF treatment.

### Gene expression profiling of cortical cultures

To confirm the cortical identity of iPSC-derived neuronal cultures used in every experiment, Trizol-extracted RNA from Day 18 vehicle-treated cultures of each genotype was profiled using a custom gene expression panel on the Nanostring platform (Nanostring Technologies). Mean negative control probe counts were subtracted from sample gene counts, before normalization using the geometric mean of six positive control probes and seven housekeeping genes (*CLTC*, *GAPDH*, *GUSB*, *PPIA*, *RPLP1*, *RPS15A* and *RPS9*).

### Cell viability assay

Live imaging of cell viability during the course of TNF treatment was performed by incubating the cultures with NUCLEAR-ID Blue/Red cell viability reagent (Enzo) diluted in culture media for 30 min, according to the manufacturer’s instructions. Cultures were washed in fresh media before images were acquired on an Opera Phenix imaging platform (Perkin Elmer) at 37°C and 5% CO_2_. For each timepoint, 37 fields of view were imaged at 20× objective in technical duplicate cultures of each treatment condition, with DAPI (ex375/em435–480) and dsRed (ex561/em570–630) laser and filter sets. For image processing, the number of red nuclei (indicating dead cells) was divided by the number of blue nuclei (indicating total cells) within each field of view to obtain a measure of the proportion of dead nuclei per condition.

### Total internal reflection fluorescence imaging

Imaging of the aggregates was performed using a purpose-built total internal reflection fluorescence microscope. The intensities of 405 nm (Oxxius Laser-Boxx, Oxxius), 488 nm (TOPTICA Photonics) and 561 nm (Cobalt Jive, Cobalt) lasers were first attenuated using neutral density filters, circularly polarized using quarter wave plates, expanded using telescopes and then passed through appropriate filters (FF01-417/60-25 for 405 nm, LL01-488-25 for 488 nm and FF01-561/14-25 for 561 nm, Semrock). The beams were then made concentric using dichroic mirrors (FF552-Di02-25x36 and FF458-Di02-25x36, Semrock) and directed into the back port of an inverted Ti-E Eclipse microscope (Nikon). The light was passed through a 1.49 N.A., 60× total internal reflection fluorescence objective. Fluorescence was collected by the same objective, separated from excitation light using dichroic mirror (Di01-R405/488/561/635, Semrock) and passed through filters appropriate for the fluorophore (BLP01-488R-25 for thioflavin T, BLP01-488R-25 for Cal-520 and LP02-568RS-25 for Cy3B, Semrock). The fluorescence was then expanded using a 2.5× relay lens and focussed onto an electron-multiplying charge-coupled device for imaging.

Aptamer DNA points accumulation for imaging in nanoscale topography (ADPAINT) and single-aggregate visualization by enhancement (SAVE) imaging was performed as described previously (Whiten *et al.*, 2018b). Briefly, round 50-mm slides first were cleaned under argon plasma (PDC-002, Harrick Plasma) for 1 h. A CultureWell coverslip (CultureWell CWCS-50R-1.0, 50 channels) was then cut in half and layered on the slide to create individual wells. Each well was then incubated in aspartic acid (1 mg/ml) for 1 h to create a surface electrostatically unfavourable to DNA binding. The chambers were then rinsed with PBS, pH 7.4, 0.02-μm filtered (Anotop25, Whatman) and the solution containing aggregates to be imaged incubated in the wells for 5 min. For ADPAINT and SAVE imaging, the cell culture media samples were first diluted 1:10 in PBS. The solution was then aspirated and replaced with imaging solution, containing 100 nM aptamer, 1 nM Cy3B-conjugated imaging strand and 5 µM thioflavin T in Tris buffer. The chambers were then sealed using another clean coverslip to prevent evaporation. The sequences of the DNA strands are provided in Table 1. All DNA strands were diluted in PBS. To prevent user bias when imaging, all images were taken in a grid using an automated script (Micro-Manager); exposure time for all frames was 50 ms. In total, 4000 frames were acquired for ADPAINT imaging; for diffraction-limited imaging (including the single-vesicle assay), 100 frames were collected. Localizations were identified using the PeakFit ImageJ plugin of the GDSC Single Molecule Light Microscopy package using a signal strength threshold of 100 and a precision threshold of 20 nm. The DBSCAN algorithm in Python 3.7 (sklearn v0.20.1) was used to identify clusters and remove spurious localizations with epsilon = 3 and minimum points threshold = 10. Aggregate lengths were calculated by skeletonization in Python 3.7 using SciPy v1.1.0. The data analysis is described in detail in Whiten *et al.* (2018b).

**Table 1:** Sequences of DNA constructs used in this work

DNA strand name	Sequence
**Aptamer + linker + docking strand**	GCCTGTGGTGTTGGGGCGGGTGCGTT ATACATCTA
**Imaging strand**	CCAGATGTAT-CY3B

### Membrane permeabilization assay

The ability of the samples to permeabilize lipid membranes was performed as described previously (Flagmeier *et al.*, 2017). This assay uses the fluorescence of Cal-520-filled vesicles immobilized on the glass coverslide to determine the relative influx of Ca^2+^ caused by the presence of protein aggregates. Cell culture media samples were used undiluted for these experiments and were incubated with the vesicles on the slides for 10 min before imaging. Antibodies/nanobodies were used to determine whether they could inhibit the permeabilization and were preincubated with the media samples for 10 min at room temperature before incubation on the slide (see Table 2 for details of nanobodies and antibodies).

**Table 2:** Antibodies and nanobodies used in single-vesicle assays

Antibody	Target	Supplier	Concentration used (nM)
**NB3**	Amyloid-β	Prof. Serge Muyldermans, Vrije Universiteit Brussel, Belgium	300
**NBSyn2**	α-Synuclein	Dr Marija Iljina, University of Cambridge, UK	300
**HT7**	Tau	Thermo Fisher Scientific (MN1000)	300
**Tau-5**	Tau	Abcam (ab3931)	300
**IgG control**	N/A	Abcam (ab6556)	300

### Statistical analysis

All statistical analyses were performed using Graphpad Prism 7. Unless otherwise stated, data were analysed with one- or two-way ANOVA, with additional *post hoc* testing performed using Dunnett’s multiple comparison in the case of significant factor effects or interactions. Data shown in Supplementary Fig. 3 were analysed using two sample Kolmogorov–Smirnov tests. Data shown in Fig. 2B and C were analysed by one-sample *t*-test, comparing washout effects to non-washout effects, which were set to a baseline of 100%. Number of independent experiments is indicated in figure legends.

### Data availability

All primary data are provided in Supplementary Table 1.

## Results

### TNF induces increased secretion of aggregates by human *PSEN1* mutant neurons

We and others have reported that human stem cell-derived cortical neurons carrying mutations in *PSEN1* that cause monogenic, familial Alzheimer’s disease alter production of extracellular Aβ to longer, more aggregation-prone forms of the peptide (Yagi *et al.*, 2011; Moore *et al.*, 2015). To study the ability of inflammation to modify the production and aggregation of pathogenic proteins, we exposed human iPSC-derived cortical neurons to two different concentrations of TNF over a prolonged treatment period. Cortical neurons from a non-demented control (Israel *et al.*, 2012) and individuals carrying two different *PSEN1* mutations (M146I and Intron 4) (Moore *et al.*, 2015) were generated from iPSCs as previously described (Shi *et al.*, 2012). These two *PSEN1* mutations were studied as they capture differing degrees of deficits in endolysosomal function, with the M146I mutation having a more pronounced phenotype, in terms of changes in lysosome size and defects in autophagy (Hung and Livesey, 2018).

**Figure 1:**
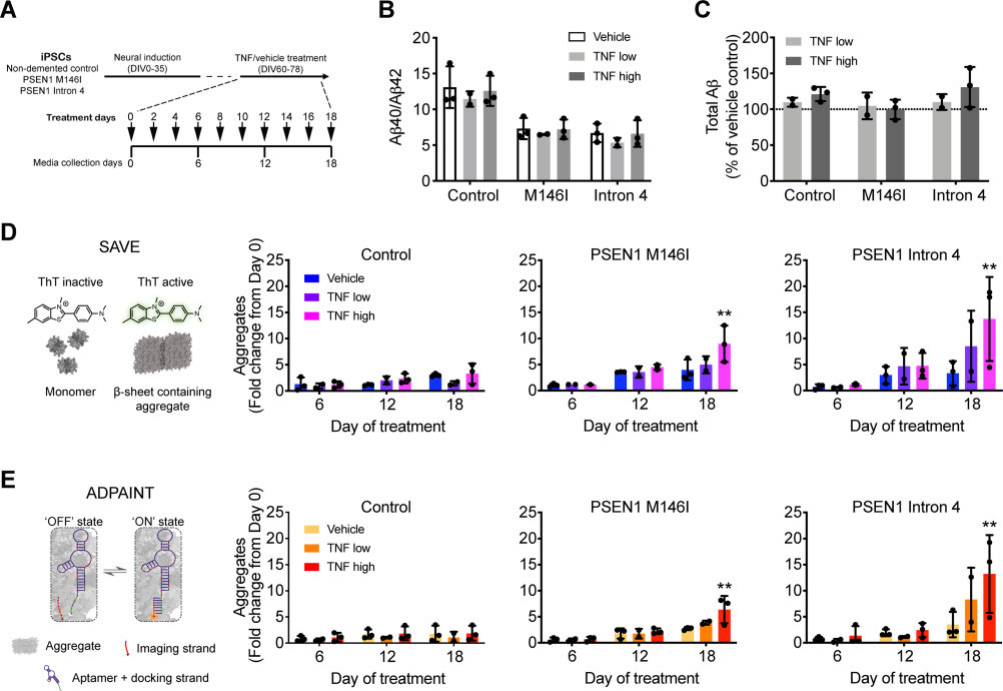
**TNF induces production of extracellular aggregates by human *PSEN1* mutant neurons.** (**A**) iPSC-derived neurons were exposed to TNF or vehicle for 18 days, with media collected every 6 days for analysis. (**B**) ELISA measurements of extracellular Aβ revealed a significant impact of genotype, but not chronic TNF treatment, on Aβ40/Aβ42 ratio (two-way ANOVA: genotype factor *F*(2,15) = 28.32, *P* < 0.0001; treatment factor *F*(2,15) = 1.024, *P* > 0.05; interaction *F*(4,15) = 0.04128, *P* > 0.05). (**C**) Similarly, there was no significant dose-dependent change in total extracellular Aβ (Aβ38 + Aβ40 + Aβ42) as a result of chronic TNF exposure (two-way ANOVA: genotype factor *F*(2,9) = 1.467, *P* > 0.05; treatment factor *F*(1,9) = 1.010, *P* > 0.05; interaction *F*(2,9) = 0.7066, *P* > 0.05). (**D**) SAVE imaging allows the imaging and quantification of individual thioflavin T-positive β-sheet-containing aggregates from neural secretomes. SAVE imaging of control secretomes revealed no effect of TNF on aggregate number (two-way ANOVA: time factor *F*(2,15) = 4.188, *P* < 0.05; treatment factor *F*(2,15) = 1.350, *P* > 0.05; interaction *F*(4,15) = 1.161, *P* > 0.05; all multiple comparisons *P* > 0.05). By contrast, high dose TNF significantly increased the number of aggregates in the secretomes of *PSEN1* M146I (two-way ANOVA: time factor *F*(2,15) = 18.60, *P* < 0.0001; treatment factor *F*(2,15) = 4.048, *P* < 0.05; interaction *F*(4,15) = 2.321, *P* > 0.05) and Intron 4 (two-way ANOVA: time factor *F*(2,15) = 7.943, *P* < 0.01; treatment factor *F*(2,15) = 2.752, *P* > 0.05; interaction *F*(4,15) = 1.536, *P* > 0.05) mutant neuronal cultures. (**E**) ADPAINT imaging uses aptamers that recognize Aβ and α-synuclein oligomers, which transiently interact with complementary imaging strands to enable detection of aggregates using super-resolution microscopy. ADPAINT imaging of the same secretomes confirmed that TNF had no effect on the production of aggregates from control neurons (two-way ANOVA: time factor *F*(2,15) = 1.217, *P* > 0.05; treatment factor *F*(2,15) = 0.8401, *P* > 0.05; interaction *F*(4,15) = 0.03294, *P* > 0.05); however, high doses resulted in a significant increase in aggregates produced by *PSEN1* M146I (two-way ANOVA: time factor *F*(2,15) = 22.38, *P* < 0.0001; treatment factor *F*(2,15) = 3.819, *P* < 0.05; interaction *F*(4,15) = 2.424, *P* > 0.05) and Intron 4 (two-way ANOVA: time factor *F*(2,15) = 11.15, *P* < 0.01; treatment factor *F*(2,15) = 2.609, *P* > 0.05; interaction *F*(4,15) = 1.838, *P* > 0.05) mutant neurons. Data in **D** and **E** are represented as a fold change in aggregate number from Day 0 baseline. *N* = 3 independent experiments in all cases. Error bars represent SD. In **D** and **E**, *P < 0.05 and **P < 0.01 versus vehicle control, Dunnett’s multiple comparisons.

Neurons were treated with either a vehicle control or TNF (0.1 and 10 ng/ml; henceforth referred to as TNF low and TNF high, respectively). Preliminary experiments found that extracellular aggregates were clearly detected after 10–12 days, and robustly increased after 16–18 days of TNF treatment, therefore, in the experiments reported here, cell culture media were replaced every 2 days and collected every 6 days for analysis (Fig. 1A). These concentrations cover the physiological range of TNF reported in the human brain in chronic (Mogi *et al.*, 1994) and acute (Waage *et al.*, 1989) neuroinflammatory conditions, although the local concentration of TNF could be higher. The cortical identity of each neuronal differentiation was confirmed by multiplexed transcriptional profiling (Supplementary Fig. 1). Data from a comprehensive single-cell RNAseq analysis of neurons of each genotype had previously found that neurons express *TNFR1*, with no detectable expression of *TNF* or *TNFR2* (Supplementary Fig. 1).

It has previously been demonstrated that TNF can act directly to increase the activity of the *APP* promoter (Ge and Lahiri, 2002), the expression of β-secretase (Yamamoto *et al.*, 2007) and the activity of γ-secretase (Liao *et al.*, 2004), resulting in increased Aβ production (Liao *et al.*, 2004; Yamamoto *et al.*, 2007). To examine the effects of TNF on the APP processing pathway in human neurons, we analysed their secretomes with multiplexed Aβ ELISAs after the 18-day treatment period. As previously reported (Moore *et al.*, 2015), the ratio of Aβ40/Aβ42 was significantly impacted by genotype, reflecting the impaired γ-secretase activity of *PSEN1* mutant neurons (Fig. 1B). However, there was no significant change in the Aβ40/Aβ42 ratio (Fig. 1B) or total Aβ (Aβ38 + Aβ40 + Aβ(42) production (Fig. 1C) in response to TNF exposure, suggesting the APP processing pathway is not altered in this system by this treatment.

In addition to the increased production of longer forms of Aβ peptides, accumulation of extracellular aggregates comprising protein and peptide oligomers is considered an important pathogenic process in Alzheimer’s disease (Hardy and Higgins, 1992; Hardy and Selkoe, 2002). Characterization of the aggregates secreted by IPSC neurons is challenging due to their low concentrations and heterogeneous size distribution, such that bulk biochemical methods cannot be used, due to insufficient sensitivity. We therefore used two complementary biophysical imaging techniques to directly study the effect of TNF exposure on neuronal production of extracellular protein aggregates. SAVE (Horrocks *et al.*, 2016) uses thioflavin T to enable the diffraction-limited imaging of β-sheet-containing aggregates in secretomes (Fig. 1D). By contrast, ADPAINT (Whiten *et al.*, 2018b) is a super-resolution technique that uses an oligonucleotide, which recognizes aggregates of Aβ and α-synuclein (TsukakoShi *et al.*, 2012) and is visualized by the transient interactions of a complementary imaging strand (Fig. 1E).

Analysis of neural secretomes using both techniques revealed no significant difference in aggregate number between control and *PSEN1* mutant neurons before TNF treatment (Supplementary Fig. 2). The limit of our resolution is 20 nm, which means that aggregates smaller than this size can still be detected but appear as 20 nm. However, the majority of the aggregates found here were larger than 20 nm and thus their size is accurately determined. We observed no significant changes in aggregate number in the secretomes of healthy control neurons following TNF exposure over the 18-day period, compared to vehicle controls, using both SAVE (Fig. 1D) and ADPAINT (Fig. 1E). In contrast, 18 days of both TNF low and high treatment induced an increase in aggregate production by both *PSEN1* M146I (4–5-fold low and 6–9-fold increase high) and Intron 4 (8–9-fold low and 13–14-fold increase high) neurons using both imaging techniques (Fig. 1D and E) with the high dose TNF treatment increase being statistically significant. These statistically significant increases in extracellular aggregates were not detected until after 18 days of treatment, indicating that this phenotype is dependent upon both the *PSEN1* mutant genotypes employed and long-term exposure to TNF.

In addition to the number of aggregates, the size and conformation of assemblies is a determinant of their potential pathogenicity (Chiti and Dobson, 2017). We therefore used ADPAINT super-resolution imaging to investigate changes in the size of individual aggregates in response to TNF exposure. In vehicle-treated cultures, we observed that control neurons produce extracellular aggregates primarily between 20 nm (the size limit of the assay) and 50 nm. *PSEN1* mutant neurons produced significantly less aggregates in this size range, instead generating larger aggregates that spanned between 50 and 150 nm (Supplementary Fig. 3A). This result correlates with our recent observations of a significant increase in large aggregates (40–200 nm) in the cerebrospinal fluid (CSF) of Alzheimer’s disease patients compared to that of healthy controls using the same assay (De *et al.*, 2019). We observed a significant reduction in the size of aggregates produced specifically by *PSEN1* mutant neurons after TNF exposure, although the magnitude of this effect size is small and does not bring the size distribution of the aggregates back in line with vehicle-treated control cultures (Supplementary Fig. 3).

Together, these data demonstrate that *PSEN1* mutant neurons produce a comparable number of extracellular aggregates to controls in normal culture conditions, although the size of aggregates from *PSEN1* mutants is significantly larger, which means that they secrete a larger mass of aggregates than controls. *PSEN1* mutant neurons specifically increase the production of extracellular aggregates in response to 18 days of TNF high exposure, with a small decrease in aggregate size observed compared to vehicle controls.

### Aggregate secretion is not reversible following TNF withdrawal and is not due to cell death

**Figure 2:**
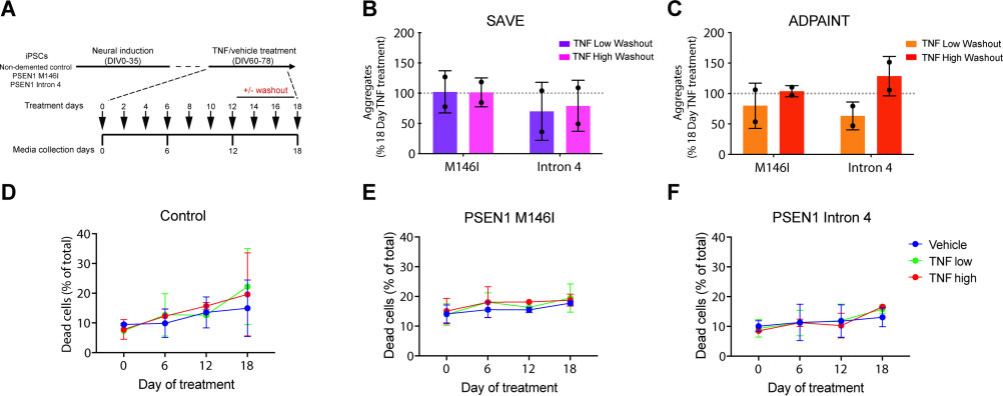
**Augmented extracellular aggregate production continues following TNF withdrawal and is not due to increased cell death.** (**A**) TNF was removed at Day 12 of treatment by switching a subset of cultures to the vehicle control, with media changed every 2 days to ensure complete washout. SAVE (**B**) and ADPAINT (**C**) imaging of secretomes subjected to TNF removal and washout at Day 12 revealed comparable number of aggregates to those cultures treated for a full 18 days (one-sample *t*-test versus 100%, *P* > 0.05 in all cases). (**D**–**F**) High content imaging revealed no significant changes in cell viability in control (**D**) (two-way repeated-measures ANOVA: time factor *F*(3,9) = 3.966, *P* < 0.05; treatment factor *F*(2,3) = 0.09118, *P* > 0.05; interaction *F*(6,9) = 0.3276, *P* > 0.05; all multiple comparisons *P* > 0.05), *PSEN1* M146I (**E**) (two-way repeated-measures ANOVA: time factor *F*(3,9) = 1.554, *P* > 0.05; treatment factor *F*(2,3) = 4.394, *P* > 0.05; interaction *F*(6,9) = 0.09461, *P* > 0.05) or Intron 4 (**F**) (two-way repeated-measures ANOVA: time factor *F*(3,9) = 3.092, *P* > 0.05; treatment factor *F*(2,3) = 0.03124, *P* > 0.05; interaction *F*(6,9) = 0.2670, *P* > 0.05) mutant neurons as a result of TNF treatment when assessed by cell viability dye. Data in **B** and **C** are represented as a percentage of the aggregate number in washout conditions at Day 18 compared with continuous 18-day TNF treatment in each case. *N* = 2 independent experiments in all assays. Error bars represent SD

As the effects of TNF on aggregate production were not acute and only observed at Day 18 of our assays, we next assessed the requirement for continuous inflammatory stress in the production of this phenotype in a series of washout experiments. Aggregates were measured in the secretomes of cultures either continuously treated with TNF for 18 days or switched to the vehicle control at Day 12 for the final 6 days, with complete media changes every 2 days (Fig. 2A). Aggregate production at Day 18 was not statistically different between neurons continuously treated with either concentration of TNF and those switched to the vehicle control at Day 12, when analysed by SAVE (Fig. 2B) and ADPAINT (Fig. 2C). Therefore, exposure of human *PSEN1* mutant neurons to TNF initiates a process that manifests even after the subsequent removal of the stimulus.

The signalling pathways of TNF in neurons are complex and have been reported to play both neuroprotective and detrimental roles (Probert, 2015). To test the possibility that extracellular aggregates were due to cell death via TNF signalling, we used a live/dead nuclear dye to quantify cell viability in cultures from each genotype across the course of the treatment by high content imaging. Exposure to either low or high doses of TNF had no significant effect on cell death in control (Fig. 2D) or *PSEN1* mutant cultures (Fig. 2E and F), indicating that reduced cell viability does not contribute to the observed increase in extracellular aggregates following TNF exposure.

### Aggregates produced by *PSEN1* mutant neurons permeabilize lipid membranes and contain Aβ and α-synuclein

**Figure 3:**
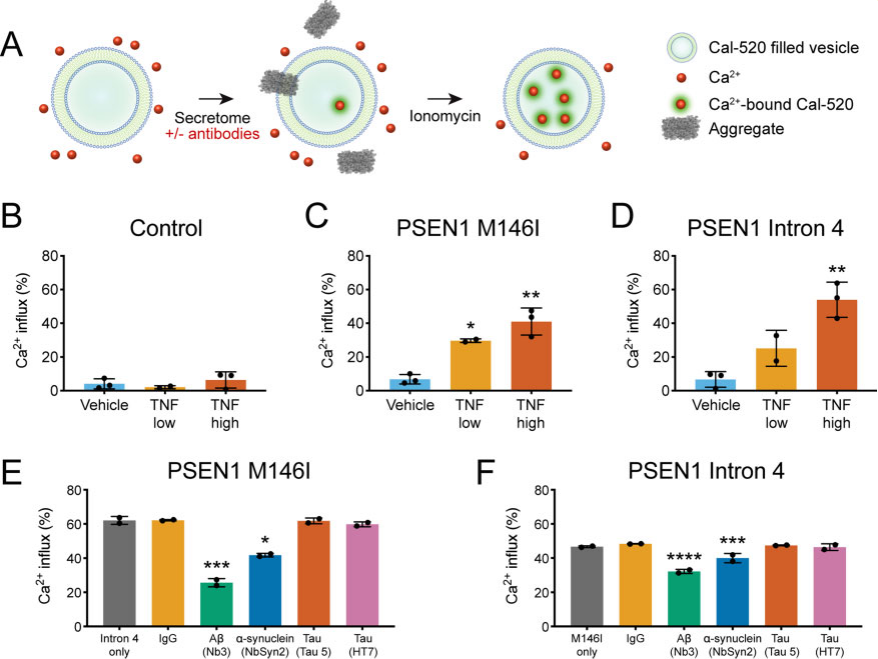
**Aggregates are composed of Aβ and α-synuclein and permeabilize lipid membranes.** (**A**) The single-vesicle permeabilization assay consists of a preparation of liposomes loaded with a fluorescent Ca^2+^ detection dye (Cal-520), which fluoresce after protein aggregates disrupt the membrane and allow the entry of Ca^2+^. Ionomycin induces maximal permeabilization and is used to benchmark liposome responses to aggregates as a percentage of total possible Ca^2+^ influx. (**B**–**D**) Exposure of liposomes to Day 18 treated neural secretomes revealed that secretomes from control neurons (**B**) did not induce permeabilization of liposomes (one-way ANOVA: *F*(2,5) = 0.8862, *P* > 0.05), but those from TNF-treated *PSEN1* M146I (**C**) (one-way ANOVA: *F*(2,5) = 30.87, *P* < 0.01) and Intron 4 (**D**) (one-way ANOVA: *F*(2,5) = 22.49, *P* < 0.01) mutant neurons induced significant liposome disruption, consistent with the presence of increased numbers of protein aggregates. (**E**, **F**) Pre-incubation of TNF high-treated neural secretomes with nanobodies to Aβ (Nb3) and α-synuclein (NbSyn2) significantly reduced the permeabilization potential of the secretomes from *PSEN1* M146I (**E**) (one-way ANOVA: *F*(5,6) = 36.25, *P* < 0.001) and Intron 4 (**F**) (one-way ANOVA: *F*(5,6) = 162.1, *P* < 0.0001) mutant neurons, while pre-incubation with control IgG or two tau-directed antibodies (Tau 5 and HT7) had no significant effect. *N* = 3 independent experiments in **B**–**D**, and *N* = 2 independent liposome preparations in **E** and **F**. Error bars represent SD. In **B**–**D**, *P < 0.05 and **P < 0.01 versus vehicle treatment, Dunnett’s multiple comparisons. In **E** and **F**, *P < 0.05, ***P < 0.001 and ****P < 0.0001 versus no antibody pre-incubation, Dunnett’s multiple comparisons

Membrane permeabilization is a primary mechanism by which extracellular protein aggregates have been hypothesized to confer toxicity (Demuro *et al.*, 2005) by allowing the entry of calcium ions leading to disrupted calcium homeostasis (Dreses-Werringloer *et al.*, 2008). We used an ultrasensitive assay to measure the membrane permeabilization activity of aggregates generated by human neurons, which we have previously validated with *in vitro* generated aggregates (Whiten *et al.*, 2018a) and those found in CSF samples from Alzheimer’s disease patients (Drews *et al.*, 2017). This assay quantifies the ability of aggregates to permeabilize the lipid bilayers of immobilized liposomes, permitting the influx of Ca^2+^ and the activation of an enclosed calcium-dependent fluorescent reporter, which is then normalized to ionomycin-mediated lysis (Fig. 3A).

Secretomes from vehicle-treated control cultures were limited in their ability to permeabilize membranes and TNF did not increase their potency (Fig. 3B). Similarly, vehicle-treated *PSEN1* M146I (Fig. 3C) and Intron 4 mutant secretomes (Fig. 3D) were relatively inert in this assay. However, lipid membrane permeabilization was significantly increased by secretomes from TNF low- and high-treated *PSEN1* M146I mutant neurons (Fig. 3C) and from TNF high-treated *PSEN1* Intron 4 mutant neurons (Fig. 3D), compared to their respective vehicle controls. These results correlate with our SAVE and ADPAINT imaging data and demonstrate that TNF treatment of *PSEN1* mutant neurons, but not non-demented controls, augments the production of extracellular aggregates that have the capacity to permeabilize lipid membranes and hence have a detrimental effect on neurons.

We have previously demonstrated that the composition of protein aggregates with activity in this assay can be directly determined by pre-incubation with candidate neutralizing nanobodies and antibodies (Drews *et al.*, 2017; De *et al.*, 2019). Using this approach, the composition of extracellular aggregates was investigated by pre-incubating the secretomes from TNF-treated *PSEN1* mutant neurons with nanobodies or antibodies to Aβ, α-synuclein and tau, all of which form aggregates in neurodegenerative diseases and are released by neurons in culture (Moore *et al.*, 2015; Evans *et al.*, 2018).

Pre-incubation with an Aβ-specific nanobody (Nb3) (Paraschiv *et al.*, 2013) significantly and substantially reduced membrane permeabilization by TNF-treated Alzheimer’s disease neuron secretomes from *PSEN1* M146I (reduction of 31.0% ± 1.9%) (Fig. 3E) and Intron 4 (reduction of 58.7% ± 2.3%) mutants (Fig. 3F). Pre-incubation with an α-synuclein-specific nanobody (NbSyn2) (Iljina *et al.*, 2017) also significantly reduced membrane permeabilization by the secretomes of TNF-treated *PSEN1* M146I (reduction of 14.2% ± 6.7%) (Fig. 3E) and *PSEN1* Intron 4 neurons (reduction of 32.7% ± 0.9%) (Fig. 3F) but by a smaller margin than Nb3. In contrast, pre-incubation with two tau-directed antibodies that bind to different epitopes had no significant effect, suggesting that tau does not form or contribute to the toxic aggregates produced under these conditions. Together, these results suggest that TNF augments the secretion of extracellular Aβ and α-synuclein-containing aggregates from *PSEN1* mutant neurons that have the capacity to permeabilize lipid membranes.

## Discussion

We report here that chronic TNF treatment of human *PSEN1* mutant neurons, but not healthy control neurons, results in increased release of toxic extracellular protein aggregates. Using single-molecule imaging techniques and membrane permeabilization assays, we determined that these aggregates contain Aβ and/or α-synuclein, potentially with other proteins, and have the capacity to permeabilize lipid membranes. We previously observed an increase in the ability of aggregates in CSF to permeabilize membranes with no accompanying change in aggregate number when comparing patients who are mildly cognitively impaired and hence at the early stages of developing Alzheimer’s disease compared to controls (De *et al.*, 2019). Furthermore, using ADPAINT, we found that this increased membrane permeabilization corresponded with an increase in the proportion of smaller aggregates present in the mildly cognitively impaired CSF, similar to the changes observed here in the aggregate size distribution on exposure of mutant neurons to TNF.

Due to the high concentration of peptide required for oligomerization, it is presumed that Aβ aggregates *in vivo* are formed intracellularly (Hu *et al.*, 2009; Friedrich *et al.*, 2010). In this study, the extracellular concentration of monomeric Aβ42 in *PSEN1* mutant secretomes was not modified by TNF treatment, and never exceeded 20 pM, which is far below the nanomolar concentrations required for fibril formation (Novo *et al.*, 2018), suggesting an intracellular origin for extracellularly detected aggregates. In addition to Aβ, we also determined that α-synuclein was an aggregate constituent, which may be explained by previous observations that Aβ42 has specific interactions with α-synuclein, which can result in bilateral aggregation of each species (Masliah *et al.*, 2001; Mandal *et al.*, 2006). The presence of Aβ and α-synuclein co-aggregates in TNF-treated *PSEN1* secretomes also cannot be excluded and has been observed *in vitro* (Iljina *et al.*, 2018).

The increased release of aggregates following TNF treatment is independent of cell death, suggesting a regulated mechanism of release from genetically vulnerable neurons. We have previously shown that in addition to altered Aβ production, neurons harbouring *PSEN1* mutations have impaired endolysosomal function, which manifests as a pronounced defect in the degradative phase of autophagy (Hung and Livesey, 2018). We hypothesize perturbed proteostasis due to altered endolysosomal trafficking and reduced autophagy may render neurons susceptible to aberrant aggregate generation and exocytosis in the presence of an additional trigger. How TNF acts as that trigger is currently not clear, but it is notable that TNF–TNFR signalling regulates autophagy in other cell types (Ge *et al.*, 2018), which may further aggravate the pre-existing defects in the autophagy-lysosomal network in *PSEN1* mutant neurons, promoting protein aggregation and release.

The aggregate size distribution that we measure for the mutant neurons, exposed to TNF, is notably similar to that we recently measured in mildly cognitively impaired and Alzheimer’s disease CSF (Supplementary Fig. S2C), using the same imaging method (De *et al.*, 2019). This suggests that similar secretion of aggregates occurs *in vivo* during the development of Alzheimer’s disease as observed from human neurons *in vitro*. Increased aggregate secretion may be a major mechanism for neurons to maintain protein homeostasis under stress, as well as a major source of Aβ monomer (Wang *et al.*, 2017).

Higher concentrations of longer protofibillar aggregates of Aβ, with sizes in the same range as detected in these experiments (40–200 nm), have previously been shown to cause an inflammatory response via TLR4, leading to production of proinflammatory cytokines including TNF (Paranjape *et al.*, 2013; Terrill-Usery *et al.*, 2016; Colvin *et al.*, 2017). A positive cycle of increased secretion of aggregates leading to increased inflammation and production of TNF and other proinflammatory cytokines could therefore drive increased production of Aβ aggregates in the Alzheimer’s disease brain. Therefore, the increase in number of Aβ aggregates that we observe in the presence of TNF treatment provides a plausible mechanism for the 3-fold increase in Aβ aggregates deposited in the Alzheimer’s disease brain over time (Roberts *et al.*, 2017). Future experiments will need to establish if the same increase in number of aggregates occurs in wild-type neurons, as well as mutant neurons, when exposed to TNF over longer times. In addition to its role as a modulator of systemic and central inflammation, TNF is a key regulator of processes in neuronal development and homeostasis, primarily through signalling via the TNF receptor 1 (TNFR1) on neurons (Santello and Volterra, 2012), which is the only TNF receptor expressed in the human iPSC-derived neurons studied here. It has been demonstrated that TNFR1 signalling can modulate synaptic strength via exocytosis of AMPA receptors (Beattie *et al.*, 2002; Stellwagen *et al.*, 2005), and endocytosis of GABA receptors (Stellwagen *et al.*, 2005), which may point to overlapping pathways involved in trafficking and exocytosis of protein aggregates in already vulnerable Alzheimer’s disease neurons in this case. Future studies will be directed towards elucidating the cellular mechanisms linking TNF signalling and the production and release of protein aggregates specifically from Alzheimer’s disease neurons.

The amyloid cascade hypothesis, which proposes that aggregation of Aβ is the central pathological event in Alzheimer’s disease pathogenesis (Hardy and Higgins, 1992; Hardy and Selkoe, 2002), has directed clinical efforts to treat the condition over the past several decades. While there have been a number of high profile clinical failures in this domain (Doody *et al.*, 2013; Selkoe, 2019), there remains significant interest in understanding the mechanisms and pathways leading to protein aggregation, which represents a shared pathogenic pathway in many neurodegenerative conditions. It is becoming accepted that in addition to protein aggregation, inflammation is a key element of early Alzheimer’s disease pathogenesis (Mhatre *et al.*, 2015). In line with this, preclinical and clinical evidence suggests that targeting inflammation—TNF in particular—is a promising therapeutic avenue. In animal models of Alzheimer’s disease, selective ablation of microglia, the primary source of proinflammatory cytokines in the CNS, or inhibition of TNF signalling, significantly reduces Aβ deposition while rescuing cognitive defects (He *et al.*, 2007; Shi *et al.*, 2011; Sosna *et al.*, 2018). Promising results from epidemiological studies and small-scale clinical trials also suggest that exposure to anti-TNF agents reduces incidence or severity of Alzheimer’s disease in humans, though there is scope for future large-scale randomized clinical trials to confirm these, at times, contradictory results (Ekert *et al.*, 2018).

Having established that TNF promotes protein aggregate production from monogenic Alzheimer’s disease neurons, which both generate longer, hydrophobic forms of Aβ and have dysfunction of their endolysosomal-autophagy network, a wide range of follow-up studies will be of interest. This includes exploring the cellular mechanisms underlying this process, the effect of other Alzheimer’s disease-associated mutations and if similar changes can be observed in the control neuron, if exposed to TNF or a combination of proinflammatory cytokines for longer times. It will also be of interest to investigate the potential proinflammatory properties of the secreted aggregates and their capability to cause LTP deficit in rodent models.

Overall, our results point to complex interactions during Alzheimer’s disease initiation *in vivo*, with early stage inflammation, either from the disease itself, or secondary insults, leading to TNF release and subsequent acceleration of the release of protein aggregates, which in turn drives further disease progression. Consequently, directly targeting these aggregates and reducing the levels of TNF and other inflammatory cytokines are both potential therapeutic targets for slowing Alzheimer’s disease pathogenesis and may be more effective when used in combination. Finally, studies of iPSC-derived neurons in the presence of chronic levels of TNF may be a more realistic model of Alzheimer’s disease than studying neurons in the absence of any cellular stress, since the secreted aggregates have similar size distributions to Alzheimer’s disease CSF and show an increase in number as observed in Alzheimer’s disease.

## Supplementary material

Supplementary material is available at *Brain Communications* online.

## Supplementary Material

fcaa146_Supplementary_Data

## Abbreviations

ADPAINT =: aptamer DNA points accumulation for imaging in nanoscale topography;
APP =: amyloid precursor protein;
Aβ =: amyloid-β;
CSF =: cerebrospinal fluid;
iPSC =: induced pluripotent stem cell;
*PSEN1* =: presenilin-1;
SAVE =: single-aggregate visualization by enhancement;
TNF =: tumour necrosis factor
