# Supplementary material for: Tumour necrosis factor induces increased production of extracellular amyloid-β- and α-synuclein-containing aggregates by human Alzheimer’s disease neurons
Source: Brain Commun. 2020 Sep 15;2(2):fcaa146. doi: 10.1093/braincomms/fcaa146 (PMC7850285; doi:10.1093/braincomms/fcaa146)
Supplement: fcaa146_Supplementary_Data [file fcaa146_supplementary_data.zip › Supplementary_figures.pdf]

A

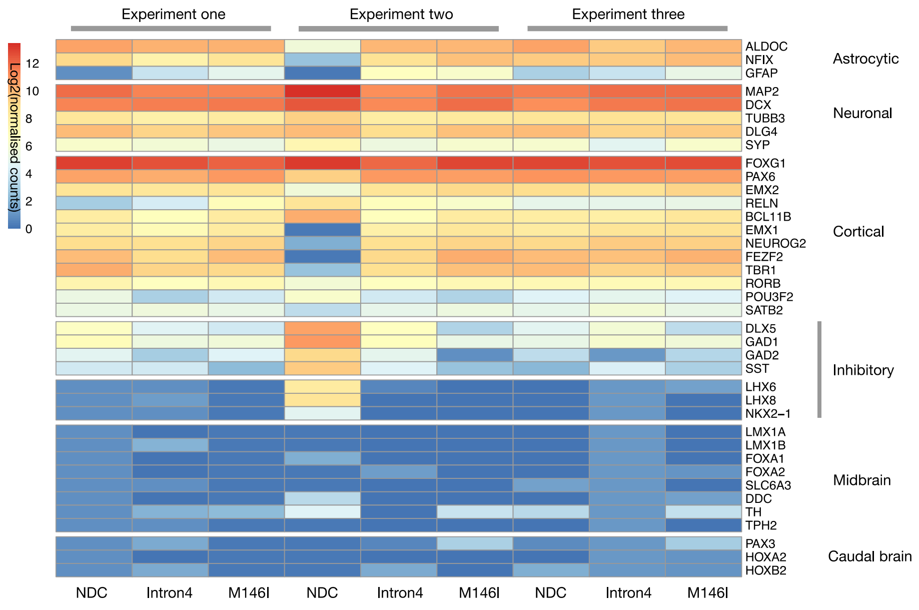

B

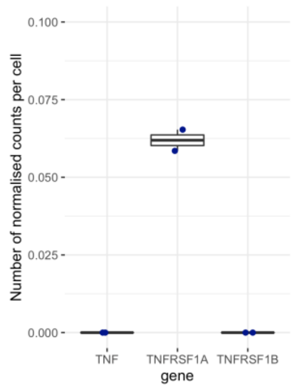

Supplementary Figure 1. Gene expression analysis confirms cortical identity of iPSC-derived neurons, and determines the expression of TNF and TNF receptors

A. Log_2 normalised counts of gene expression of all neural inductions used in this study indicate a high level of expression of neuronal and cortical genes, low levels of expression of midbrain and caudal brain genes, and, with the exception of one non-demented control (NDC) induction in experiment two, a low level of expression of inhibitory neuronal markers across all tested genotypes. NDC = Non-demented control, Intron4 = *PSEN1* Intron 4 mutant, M146I = *PSEN1* M146I mutant.

B. Normalised single cell expression values of TNF and the two TNF receptors, TNFRSF1A and TNFRSF1B in human iPSC-derived neurons. TNFRSF1A is expressed in neurons, whereas TNF and TNFRSF1B are not detectably expressed. N = 2 independent neural inductions.

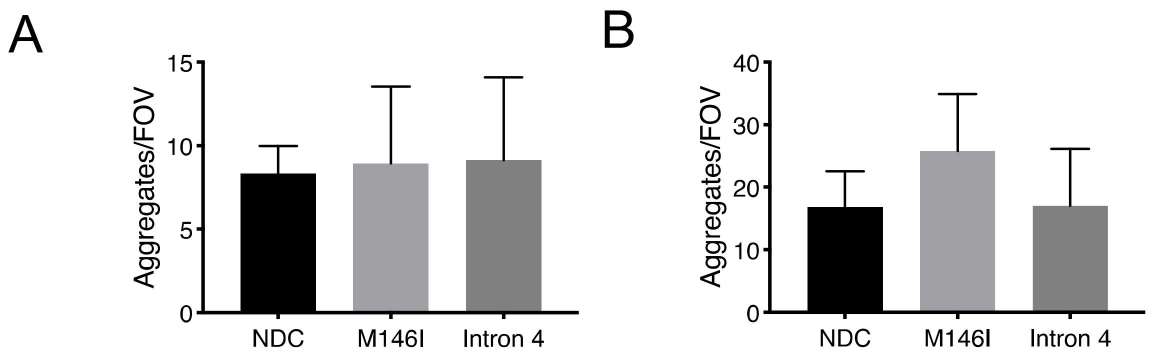

Supplementary Figure 2. Number of extracellular aggregates is similar between genotypes at baseline

SAVE (A) and ADPAINT (B) imaging of extracellular aggregates produced by iPSC-neurons reveals no significant difference in aggregate number between control and AD neurons prior to TNF treatment (One-way ANOVA). FOV = Field of view. N = 3 independent experiments. Error bars represent S.D.

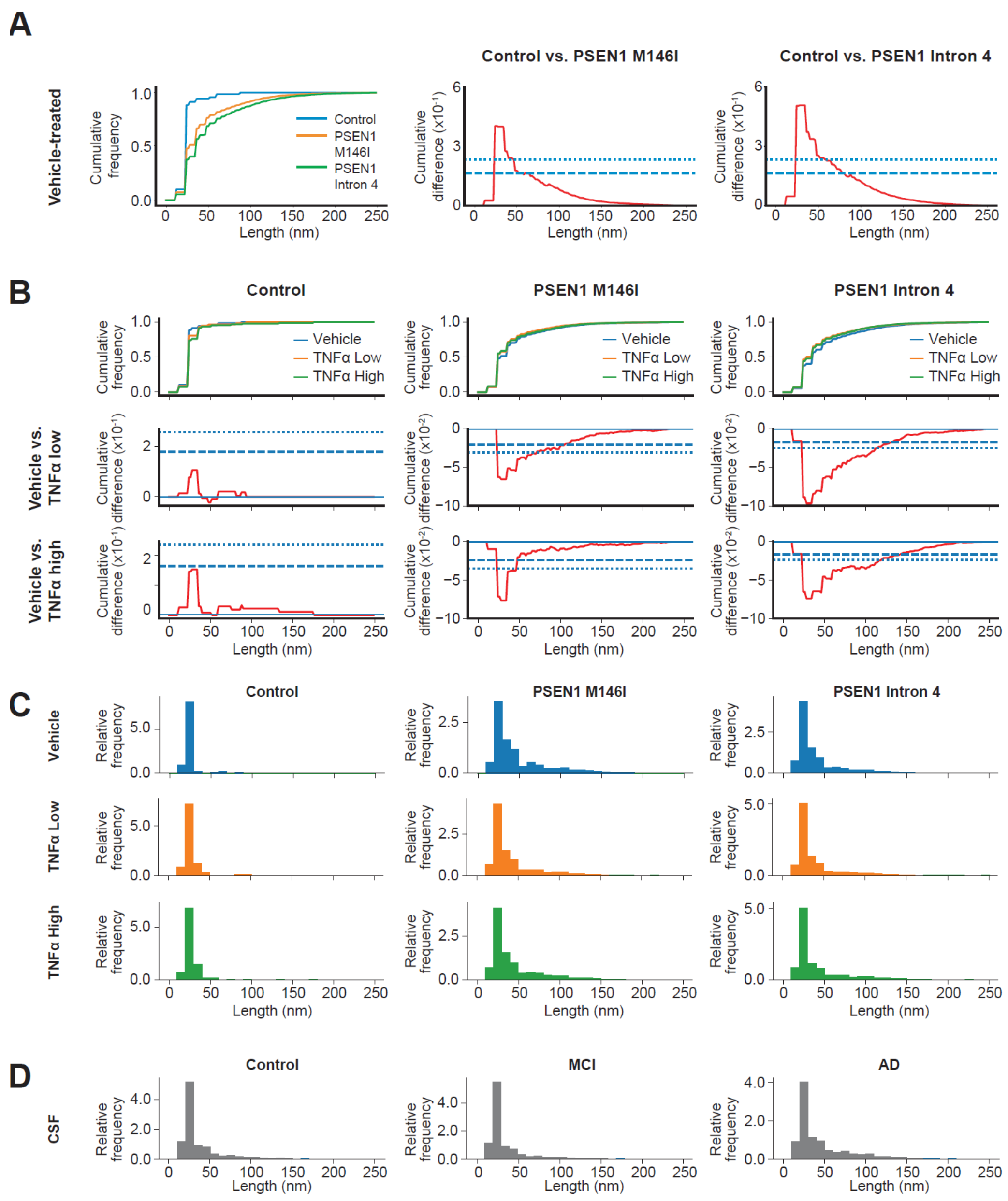

Supplementary Figure 3. Extracellular aggregates produced by *PSEN1* mutant neurons are larger than those produced by control neurons, are similar in size to aggregates in human AD CSF, and are reduced in size by TNF exposure

(A) As measured by ADPAINT super resolution imaging, control neurons produce extracellular aggregates primarily between 20 and 50 nm in size, whereas *PSEN1* mutant neurons produce significantly less aggregates in this size range, instead producing larger aggregates between 50 and 150 nm in size. (B, C) After treatment with TNF, *PSEN1* mutant, but not control, neurons

produce significantly more aggregates in the 20 – 50 nm range when compared to vehicle treatment, although the size distribution still differs from that of aggregates produced by control neurons. (D) When compared with previously published data from our group (Acta Neuropathol Commun. 2019,7(1):120. doi: 10.1186/s40478-019-0777-4), the size distribution of aggregates produced by *PSEN1* mutant neurons is similar to those observed in human AD CSF. Dotted lines represent the 95% confidence interval, and dashed lines represent the 99% confidence interval of a two-sample Kolmogorov-Smirnov test comparing *PSEN1* mutant neuron-derived aggregate profiles to the control neuron-derived aggregate profile (A) and TNF-treated aggregate profiles to vehicle treated profiles (B). Data are representative of three independent experiments.
